# Supplementary material for: Magnetism and topology in Tb-based icosahedral quasicrystal
Source: Sci Rep. 2021 Sep 3;11:17679. doi: 10.1038/s41598-021-97024-w (PMC8417217; doi:10.1038/s41598-021-97024-w)
Supplement: Supplementary file 1 — Supplementary Information. [file 41598_2021_97024_MOESM1_ESM.pdf]

Supplementary Information for
“ Magnetism and topology in Tb-based icosahedral quasicrystal ”

Shinji Watanabe

Department of Basic Sciences, Kyushu Institute of Technology,

Kitakyushu, Fukuoka 804-8550, Japan

(Dated: July 31, 2021)

I. CRYSTALLINE ELECTRIC FIELD HAMILTONIAN

Recently, the crystalline electric field (CEF) Hamiltonian in the rare-earth based quasicrystal (QC) and approximant crystal (AC) is formulated by operator equivalents [1] as

$$H_{\rm CEF} = \sum_{\ell=2,4,6} \left[B_\ell^0(c) O_\ell^0(c) + \sum_{\eta=c,s} \sum_{m=1}^{\ell} B_\ell^m(\eta) O_\ell^m(\eta) \right], \tag{1}$$

where $O_\ell^m(c)$ and $O_\ell^m(s)$ are the Stevens operators [2] with ℓ being the azimuthal quantum number and m being the magnetic quantum number. In Eq. (1), the coefficient $B_\ell^m(\eta)$ is defined as

$$B_\ell^m(\eta) = -|e| C_\ell^m \langle r^\ell \rangle \alpha_\ell h_\ell^m(\eta). \tag{2}$$

Here, C_ℓ^m is given by $C_2^0 = \sqrt{\pi/5}$, $C_2^1 = 2\sqrt{6\pi/5}$, $C_2^2 = \sqrt{6\pi/5}$, $C_4^0 = \sqrt{\pi}/12$, $C_4^1 = \sqrt{5\pi}/3$, $C_4^2 = \sqrt{10\pi}/6$, $C_4^3 = \sqrt{140\pi}/6$, $C_4^4 = \sqrt{70\pi}/12$, $C_6^0 = \sqrt{13\pi}/104$, $C_6^1 = \sqrt{546\pi}/52$, $C_6^2 = 4\sqrt{5460\pi}/793$, $C_6^3 = \sqrt{5460\pi}/104$, $C_6^4 = 3\sqrt{182\pi}/104$, $C_6^5 = \sqrt{9009\pi}/52$, and $C_6^6 = \sqrt{12012\pi}/208$ [1]. The Stevens factors for Tb^{3+} are given as $\alpha_2 = -1/99$, $\alpha_4 = 2/16335$, and $\alpha_6 = -1/891891$ [3]. The Dirac-Fock calculation for Tb^{3+} yeilds $\langle r^2 \rangle = 0.2302$ Å^2, $\langle r^4 \rangle = 0.1295$ Å^4, and $\langle r^6 \rangle = 0.1505$ Å^6 [4].

In Eq. (2), $h_\ell^m(c)$ is given by

$$h_\ell^m(c) = {\rm Re} \left[\sum_{i=1}^{16} \frac{q_i}{R_i^{\ell+1}} (-1)^m Y_\ell^m(\theta_i, \varphi_i) \right], \tag{3}$$

where $Y_\ell^m(\theta, \varphi)$ is the spherical harmonics. The coefficient $B_\ell^m(s)$ is obtained by inputting $h_\ell^m(s)$ defined by

$$h_\ell^m(s) = {\rm Im} \left[\sum_{i=1}^{16} \frac{q_i}{R_i^{\ell+1}} (-1)^m Y_\ell^m(\theta_i, \varphi_i) \right] \tag{4}$$

into Eq. (2) [1].

Since in the Tsai-type cluster shown in Fig. 1 in the main text, the occupancy of the cluster center is small (e.g. 23% in $Au_{70}Si_{17}Tb_{13}$ [6]), we neglect the cluster center (see Fig. 1a in the main text). We note that considering the contribution from the cluster center and the Si/Au mixed sites in the CEF Hamiltonian (1) may cause a slight change from the θ dependence of α in Fig. 2c in the main text. Moreover, depending on the compositions of Au-SM-R with chemical disorder of the Au/SM sites, the α dependence of θ can also change slightly from Fig.2c. To reflect these effects, we show the J_2/J_1-θ phase diagram with the

wide range of θ in Fig.3a in the main text, which is relevant generally in the Tb-based ACs and QCs.

II. HEDGEHOG STATE

The hedgehog state is the magnetic texture where all the magnetic moments at the Tb sites on the icosahedron (IC) are directed outward. We find that the hedgehog state is stabilized in the wide-θ region for $0 \le \theta \le 23^\circ$ in Fig. 3a in the main text. It is noted that the hedgehog state for $\theta = 0^\circ$ has also been reported to appear in the spin-1 Heisenberg model on the IC [5]. By considering the effect of the CEF, we have shown that the hedgehog state is stabilized in the realistic CEF-parameter region ($\alpha \sim 4$) in Fig. 3a. Here we show the hedgehog states for finite θ in Fig. S1.

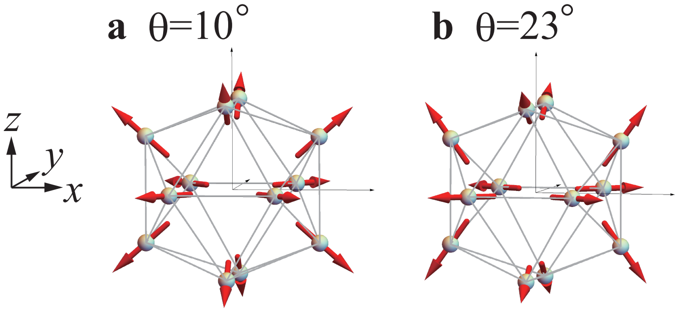

FIG. S1. (color online) **a**, The hedgehog state for $\theta = 10^\circ$. **b.**, The hedgehog state for $\theta = 23^\circ$. (This figure is created by using Adobe Illustrator CS5 Version 15.1.0.)

We confirmed that uniform distribution of the hedgehog state for finite θ are also stabilized in the QC. By applying the model (1) to the $Cd_{5.7}Yb$-type QC with the N.N. interaction J_1 and the N.N.N. interaction J_2 for the intra IC and inter IC as described in the main text, we confirmed that the uniform distribution of the hedgehog is stabilized in the region denoted by red squares in Fig. 3a in the main text, suggesting the long-range order. Figure S2 shows the hedgehog order for $\theta = 23^\circ$ in the QC.

III. ANTIWHIRLING STATE

The antiwhirling state is shown in Fig. 3b (orange square for $\theta = 90^\circ$) in the main text. Here the antiwhirling state seen from the (111) direction is shown in Fig. S3. On the top triangular surface, the magnetic moments at the three vertices swirl in the clock-wise direction. On the middle honeycomb surface, the magnetic moments at the six vertices swirl in

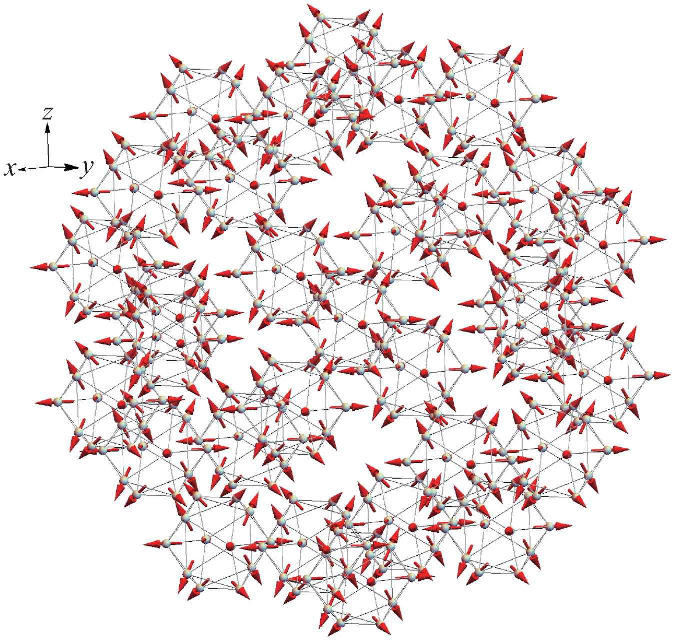

FIG. S2. (color online) The long-range order of the hedgehog state for $\theta = 23°$ in the QC. (This figure is created by using Adobe Illustrator CS5 Version 15.1.0.)

the anticlock-wise direction. On the bottom triangular surface, the magnetic moments at the three vertices swirl in the clock-wise direction. Since the topological charge calculated for all the triangular surfaces in the IC for this state is $n = -3$, this state is called antiwhirling state, which is regarded as the emergent antimonopole with the "charge" −3. The whirling state where all the magnetic moments are inverted (see Fig. 3e in the main text) is characterized by the topological charge $n = 3$, which is regarded as the emergent monopole with the "charge" +3.

It is noted that the whirling-moment state has actually been observed in the AC $Au_{72}Al_{14}Tb_{14}$, where the magnetic moment at Tb is lying in the mirror plane with the angle $\theta = 86°$ from the pseudo 5-fold axis (see Fig.1f in the main text) [7].

By applying the model (1) in the main text to the $Cd_{5.7}Yb$-type QC, we find that uniform distribution of the antiwhirling state is realized when the antiferromagnetic next-nearest-neighbor interaction is dominant in terms of the inter-IC interaction. We show the uniform arrangement of the antiwhirling state for $\theta = 90°$ in Fig. S4. Here, we set the parameters in the model (1) as $J_1 = -1$ and $J_2 = -0.1$ for the intra IC and $J_1^{\rm inter} = 0$ and $J_2^{\rm inter} = -0.1$ for the inter IC.

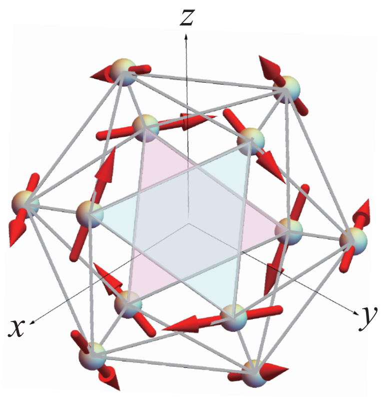

FIG. S3. (color online) The antiwhiling state seen from the (111) direction. Top (bottom) surface of triangle is illustated by light blue (pink). (This figure is created by using Adobe Illustrator CS5 Version 15.1.0.)

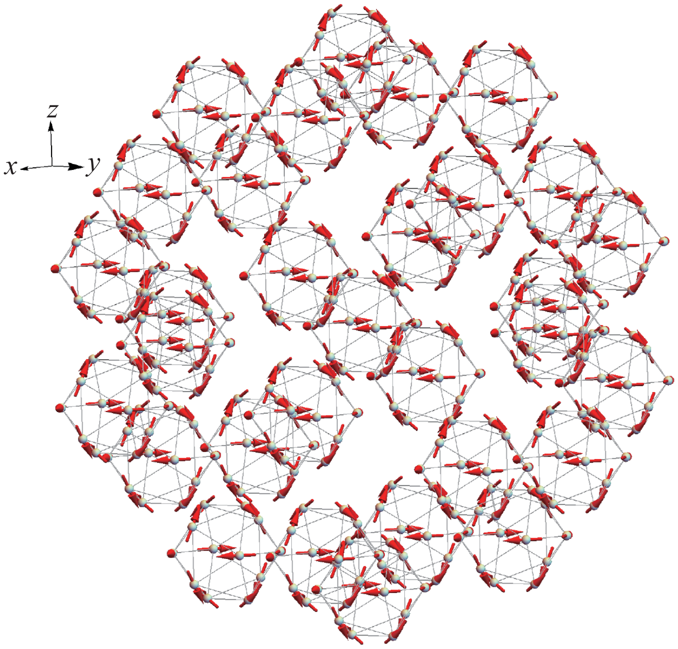

FIG. S4. (color online) The long-range order of the antiwhiling state for $\theta = 90^{\circ}$ in the QC. (This figure is created by using Adobe Illustrator CS5 Version 15.1.0.)

[1] Watanabe, S. & Kawamoto, M., Crystalline electronic field in rare-earth based quasicrystal and approximant: analysis of quantum critical Au-Al-Yb quasicrystal and approximant, *J. Phys. Soc. Jpn.* **90**, 063701 (2021).

[2] Stevens, K. W. H., Matrix elements and operator equivalents connected with the magnetic properties of rare earth ions, *Proc. Phys. Soc. A* **65**, 209 (1952).

[3] Hutchings, M. T., Point-charge calculations of energy levels of magnetic ions in crystalline electric fields, *Solid State Physics* **16**, 227 (1964).

[4] Freeman A. J. & Desclaux, J. P., Dirac-Fock studies of some electronic properties of rare-earth ions, *J. Magn. Magn. Mater.* **12**, 11 (1979).

[5] Suzuki, S., Tamura, R. & Sugimoto. T., Classical and quantum magnetic ground states on an icosahedral cluster, *Mater. Trans.* **62**, 367 (2021).

[6] Hiroto, T. *et al.*, Noncoplanar ferrimagnetism and local crystalline-electric-field anisotropy in the quasicrystal approximant $Au_{70}Si_{17}Tb_{13}$, *J. Phys.: Condens. Matter* **32**, 415802 (2020).

[7] Sato, T. J. *et al.*, Whirling spin order in the quasicrystal approximant $Au_{72}Al_{14}Tb_{14}$, *Phys. Rev. B* **100**, 054417 (2019).
